# Immunohistochemical Profile and 47-Gene Next-Generation Sequencing (NGS) Solid Tumor Panel Analysis of a Series of 13 Strumal Carcinoids

**DOI:** 10.1007/s12022-020-09608-3

**Published:** 2020-03-02

**Authors:** S. Theurer, M. Ingenwerth, T. Herold, K. Herrmann, K. W. Schmid

**Affiliations:** 1 Institute of Pathology, University Hospital Essen, University of Duisburg-Essen, Hufelandstr, 55, 45147 Essen, Germany; 2 Institute of Nuclear Medicine, University Hospital Essen, University of Duisburg-Essen, Essen, Germany

**Keywords:** Strumal carcinoid, Struma ovarii, Somatostatin receptor, SSTR, Neuroendocrine tumor

## Abstract

Strumal carcinoid is an extraordinary rare tumor of the ovary consisting of thyroid tissue intermixed with neuroendocrine tumor component. The cellular origin of strumal carcinoids has been an area of debate. There is also little data on detailed immunohistochemical and molecular characteristics of these neoplasms. For this reason, this series investigated the characteristics of a series of 13 strumal carcinoids using immunohistochemical markers and a 47-gene next-generation sequencing (NGS) solid tumor panel analysis. Both cellular components showed thyroglobulin expression in all tumors. TTF-1 expression was noted in both cellular components of 11 cases. Chromogranin A was positive in both components of most tumors (*n* = 12, 92.3% in the neuroendocrine component and *n* = 10, 76.9% in the thyroid follicular component). Synaptophysin stained the neuroendocrine component of all cases, and it was also identified in the follicular thyroid component of a single case. All tumors were negative for CDX2 and calcitonin. ISLET1 was positive in the neuroendocrine component of 8 cases (6.5%). With the exception of one case, all tumors were positive for SSTR2a. The tumors were associated with a low Ki67 labeling index. All cases were microsatellite stable and no pathogenic mutations were identified using a 47-gene NGS solid tumor analysis. This series underscored that strumal carcinoids are distinct neuroendocrine tumors. The synchronous expression for thyroid follicular epithelial and neuroendocrine differentiation biomarkers may suggest a precursor cell origin displaying mixed-amphicrine differentiation. While strumal carcinoids can be diagnosed by their typical morphology and immunohistochemical profile, frequent SSTR expression may serve as a potential theranostic biomarker in the management of affected patients. In addition, the absence of common driver mutations in the NGS solid tumor panel may suggest that these neoplasms seem to be genetically unrelated to follicular epithelial–derived thyroid tumors and potentially different than other commonly identified well-differentiated neuroendocrine neoplasms. Therefore, further studies focusing on molecular characteristics of this entity are still needed.

## Introduction

Carcinoid tumors of the ovary are rare and account for less than 1% of all malignant neoplasm of the ovary [1]. They are subclassified into 4 types: insular, mucinous, trabecular, and strumal. Among these, strumal carcinoids are extremely rare and are composed of thyroid tissue admixed with neuroendocrine tumor displaying insular or trabecular growth. These can reach a large tumor size and can be seen in association with elements of teratoma [2]. Several case reports identified that patients can manifest with severe obstipation due to the antiperistaltic effect of peptide YY, which seems to be frequently produced by tumor cells [3–5].

The cellular origin of strumal carcinoids has been an area of discussion since their first description in the 1970s. It seems to be proven that these are pure neuroendocrine tumors, as both neoplastic components, histologically presenting as “follicular thyroid” and “insular/trabecular neuroendocrine” components, contained neurosecretory granules [6, 7]. In addition, an origin of C-cell was also postulated given immunohistochemical calcitonin expression in some cases [8]. The vast majority of strumal carcinoids are biologically indolent and curable by oophorectomy [1]. Only very few cases with metastatic disease were described [9, 10]. Only one tumor-associated death has been published [11]. Due to the rarity of strumal carcinoids, knowledge about this tumor entity has been mostly generated from case reports [1, 12, 13]. In addition, a large series of 50 cases was published in 1980 addressed to the description of macroscopic and microscopic architectural features as well as the clinical outcome [11]. Expression of somatostatin receptors (SSTR) 2a and 5 is common in most well-differentiated neuroendocrine tumors [14] and these have been served as theranostic biomarkers [15]. Although SSTR expression was reported in a previous report of strumal carcinoid, the expression profile of SSTRs is not systematically investigated in these neoplasms. In addition, virtually nothing is known about molecular genetic alterations of strumal carcinoids. For instance, it is unknown if these tumors harbor similar alterations as seen in thyroid neoplasms and/or other neuroendocrine neoplasms. In light of the knowledge gap, this series investigated the characteristics of a series of 13 strumal carcinoids using immunohistochemical (bio)markers and a 47-gene next-generation sequencing (NGS) solid tumor panel analysis.

## Material and Methods

We reviewed a case series of 13 strumal carcinoids, collected from surgery material in Germany between 2008 and 2019 and send to us for second opinion. Every case was seen and diagnosed by two specialists for thyroid and neuroendocrine pathology (KWS and ST). The (female) patients presented with a median age of 52.07 years (range 26–78 years). Tumor size varied from 0.3 to 4.2 cm (median size 1.35 cm).

### Immunohistochemical Staining

We performed immunohistochemical staining for thyroglobulin (DAKO, clone polyclonal rabbit, dilution 1:20000), TTF-1 (Roche, clone 8G7G3/1 mouse, RTU), chromogranin A (Leica, clone 5H7, dilution 1:100), synaptophysin SP11 (Cell Marque, clone MRQ-40 rabbit, RTU), Ki67 (Roche, clone 30-9, RTU), ISLET1 (Abcam, clone polyclonal rabbit, dilution 1:1000), CDX2 (Zytomed, clone EPR2764, dilution 1:500), calcitonin (DAKO, clone polyclonal rabbit, dilution 1:30000), SSTR2a (Zytomed, clone polyclonal rabbit, dilution 1:50), and SSTR5 (Zytomed, clone polyclonal rabbit, dilution 1:40). All stainings were performed on the Ventana Benchmark Ultra System.

### Next-Generation Sequencing

Multiplex PCR and purification were performed with the GeneRead DNAseq Custom Panel V2, GeneRead DNAseq Panel PCR Kit V2 (QIAgen), and Agencourt® AMPure® XP Beads (Beckmann). A total amount of 45 ng DNA was used to perform multiplex PCR (four primer pools with 10 ng/primer pool+ 10% excess volume). Library preparation was performed using NEBNext Ultra DNA Library Prep Set for Illumina (New England Biolabs; NEB), according to the manufacturer’s recommendations. The pooled library was sequenced on MiSeq (Illumina; 2 × 150 bases paired-end run) and analyzed by the Biomedical Genomics Workbench (CLC Bio, QIAgen). Within the Biomedical Genomics Workbench, demultiplexed paired-end sequencing data was mapped to human genome (version hg19). A local realignment was performed to reach better alignment quality, especially for regions with small insertions or deletions. All reads which were mapped outside of targeted regions were deleted after the mapping process. In a filtering step, all reference variants and variants found in dbSNP common, 1000 genome project, and HapMap were deleted. An allele frequency of minimum 5% and coverage of at least 50 mapped reads were applied. For targeted sequencing, a customized panel was designed containing regions of interest. The customized panel included 47 genes. The analyzed genes and exons are listed in Table 1. The regions were covered by a total of 2193 amplicons and the total length of targeted regions was 176,370. For all samples, an average coverage of approximately 2200× was obtained.

**Table 1:** Genes and exons included in NGS analysis

Gene	Exons	Gene	Exons
AKT1	All	MAPK3	All
AKT2	All	MDM2	All
ARID1A	All	MET	3, 8, 11, 14, 19
ARID1B	All	MLH1	All
ATM	All	MSH2	All
BAP1	All	NF1	All
BCLAF1	All	NRAS	2.4
BRAF	11.15	PALB2	All
BRCA1	All	PBRM1	All
BRCA2	All	PDGFRa	12, 14, 18
EGFR	18–21	PIK3CA	3, 5, 10, 16, 21
ERBB2	5, 6, 15, 20, 23, 29	PTEN	All
GNA11	All	RAF1	All
GNAQ	All	RNF43	All
GNAS	All	RPA1	All
IDH1	4	SF3B1	14, 15, 16
IDH2	4	SMAD4	All
KDM6A	All	SMARCA2	All
KIT	9–11, 13, 17, 18	SMARCA4	All
KRAS	2.4	SMARCB1	All
MAP2K1	All	STK11	All
MAP2K2	All	TP53	All
MAPK1	All	TSC1	All
		TSC2	All

### Microsatellite Analysis

DNA was isolated from FFPE Material and the Microsatellite Instability Analysis System (Promega) for Microsatellite Markers BAT-25, BAT-26, NR-21, NR-24, and MONO-27 was performed and analyzed with a 3500 Genetic Analyzer according to manufacturer’s protocol (Applied Biosystems).

## Results

### Histomorphology

**Fig. 1:**
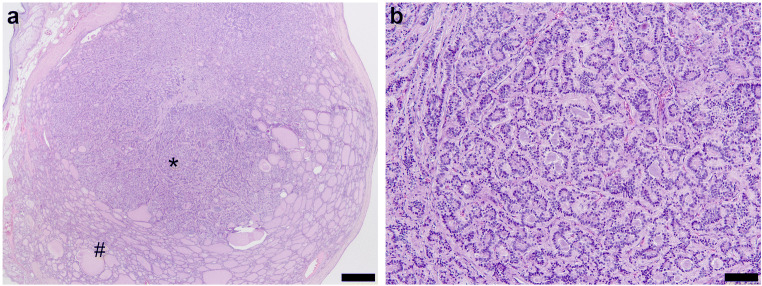
Strumal carcinoid is composed of two distinct components. **a** Neoplastic thyroid-like tissue (#) intermixed with tumor areas showing classical features of neuroendocrine tumors (*), HE × 20, scale bar 500 μm. **b** Tumor cells of the neuroendocrine component show monomorphic round-shaped nuclei with trabecular- and rosette-like architecture, HE × 100, scale bar 100 μm

All tumors included in the series were composed of normal-appearing “thyroid tissue” with follicular architecture admixed with varying amount of trabecular, insular, or nested areas resembling well-differentiated neuroendocrine tumors of other origins (Fig. 1). The nuclei of the neuroendocrine tumor component were round and enlarged with a characteristic pepper-and-salt chromatin pattern. No necrosis or mitotic activity was detected.

### Immunohistochemistry

**Fig. 2:**
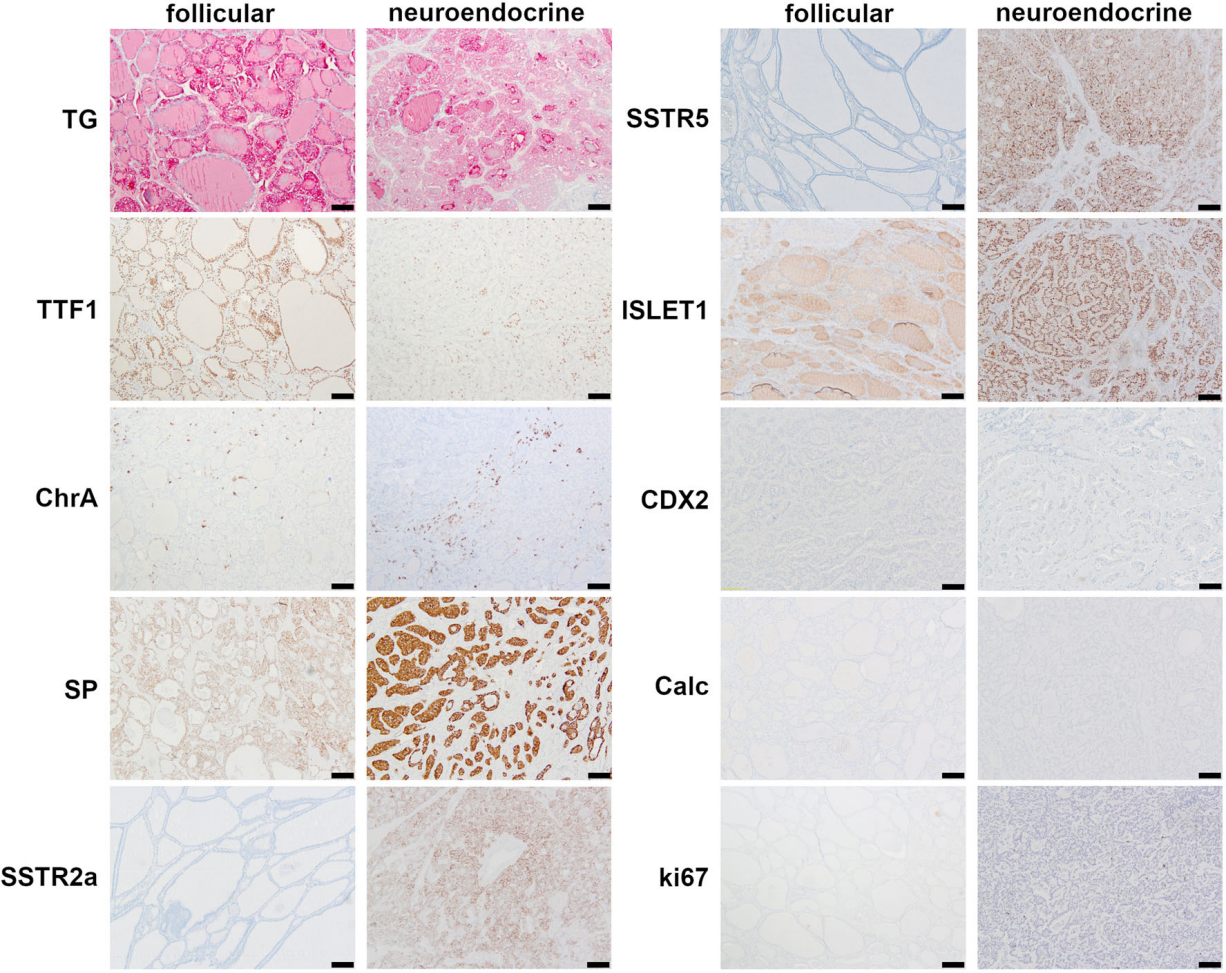
Immunohistochemical staining for different antibodies in both components of strumal carcinoid: follicular tumor component on the left and tumor areas resembling neuroendocrine tumor on the right side of the figure (TG = thyroglobulin, ChrA = chromogranin A, SP = synaptophysin, Calc = calcitonin). Magnification × 100, scale bar 100 μm

Immunohistochemical staining was evaluated for both components separately. Both cellular components showed thyroglobulin expression in all tumors. TTF-1 expression was noted in both cellular components of 11 (84.6%) cases. Chromogranin A was also positive in both components of most tumors (*n* = 12, 92.3% in the neuroendocrine component and *n* = 10, 76.9% in the thyroid follicular component). Synaptophysin stained the neuroendocrine component of all cases, and it was also identified in the follicular thyroid component of a single case (7.7%). Twelve cases had a Ki67 proliferation index of < 2%. One case had a Ki67 proliferation index of 5%. No mitotic figures are noted. All tumors were negative for CDX2 and calcitonin. Staining for ISLET1 showed positive nuclear immunostaining exclusively in the neuroendocrine component of 8 cases (6.5%). SSTR2a was immunohistochemically detected in the neuroendocrine component of 12 out of 13 cases (92.3%). Five cases were also immunoreactive for SSTR5 (41.7%) in the neuroendocrine component. The “thyroid-like” tumor component was completely negative for both SSTRs in all cases. Only one case was negative for both SSTR2a and SSTR5. Details on staining results are listed in Table 2 and displayed in Fig. 2.

**Table 2:** Expression of different immunohistochemical markers, divided according to the two different tumor components of strumal carcinoid

Immunohistochemical marker	Positive staining result, *n* (%)	
	Follicular component	Neuroendocrine component
Chromogranin	10 (76.9)	12 (92.3)
Synaptophysin	1 (8.3)	13 (100)
Thyroglobulin	13 (100)	13 (100)
TTF1	11 (84.6)	10 (84.6)
ISLET1	9 (69.2)	5 (38.4)
CDX2	0	0
Calcitonin	0	0
SSTR2a	0	12 (92.3)
SSTR5	0	6 (46.1)

### DNA-Sequence Analyses of Microsatellites

Sequence analyses of the microsatellites NR-21, BAT-26, BAT-25, NR-24, and MONO-27 revealed stable conditions in all cases.

### Next-Generation Sequencing

Molecular genetic investigations via next-generation sequencing with 47 genes, 2193 amplicons, and a total length of targeted regions of 176,370 (see Table 1) did not detect any known or clinically relevant mutations.

## Discussion

Strumal carcinoids are extraordinary rare tumors. Our morphological and clinical knowledge is mainly based on case reports, which were published over the last five decades in the literature. Since its first description in 1970, only one larger series of 50 cases analyzed architectural features of this disease. We hereby present the first large series of 13 cases of strumal carcinoids focusing on immunohistochemical biomarker expression related to thyroid follicular epithelial cells and neuroendocrine differentiation along with a 47-gene NGS solid tumor panel analysis.

Histomorphological features indicated that these tumors consist of a “normal thyroid-like” component and a neuroendocrine component, which raised the question whether strumal carcinoids are distinct tumors themselves or two separate tumors in which the neuroendocrine tumor represents a somatic malignancy arising in the background of ovarian teratoma with thyroid follicular epithelial proliferation. There is no comparable type of primary tumor in the thyroid. Even the very rare mixed medullary and follicular carcinoma cannot be compared with this entity since the follicular epithelial component consists of either papillary thyroid carcinoma or follicular thyroid carcinoma [2].

Earlier investigations questioned the origin of strumal carcinoids. Interestingly, different studies came up with different conclusions. In some ultrastructural studies, cytoplasmic neurosecretory granules suggested these neoplasms as a pure neuroendocrine tumor with follicular structures [6, 7]. Several subsequent studies argued against these considerations and showed thyroglobulin expression in both parts of the tumor or at least in transition between follicular and insular structures [16, 17]. Hamazaki et al. showed TTF-1 positivity only in follicular parts of the tumor in 2 cases, and concluded that the nested “neuroendocrine” part of the tumor has no thyroidal differentiation [18]. In this series, we also detected thyroid differentiation markers (thyroglobulin and TTF-1) and neuroendocrine markers (chromogranin and synaptophysin) in both cellular components. These findings may support the theory of a tumor arising from a single precursor cell, harboring mixed-amphicrine features of both follicular epithelial cell of the thyroid (e.g., immunohistochemical features) and neuroendocrine cells (e.g., neurosecretory granules in electron microscopy, various neuroendocrine markers). One can hypothesize that the mixed morphology may occur during tumorigenesis represented by a thyroid-like follicular and a trabecular/insular/solid neuroendocrine-like growth pattern. An origin of C-cells was also considered for these tumors based on calcitonin reactivity in some reported cases [19, 20]. Our thorough immunohistochemical analysis failed to show any calcitonin expression in these neoplasms. Although commercially available calcitonin antibodies in the early years may have showed nonspecific results, we cannot exclude that in some cases a few cells may express calcitonin as seen in other neuroendocrine neoplasms. However, it seems to be highly unlikely that strumal carcinoids represent a tumor of C-cell origin or even a medullary thyroid carcinoma arising within a struma ovarii. These considerations can also be supported by the fact that C-cells do not express thyroglobulin.

In previous reports, peptide YY was detected (a peptide hormone produced by neuroendocrine cells of distal small intestine and most L-cells that are seen in the gastrointestinal tract and enriched in the colon) in strumal carcinoids. This has led to the assumption that the origin of strumal carcinoids may be of midgut- or hindgut-derived neuroendocrine precursor cells. The majority of neuroendocrine cells of the small intestine usually express CDX2 [21]. While this series did not assess peptide YY expression in these tumors, negative CDX2 expression in the current series may argue against the origin of midgut-derived neuroendocrine cells.

As described in former case reports [22], this series also confirmed a frequent expression rate of SSTRs (especially SSTR2a) in strumal carcinoids. Only one single case failed to show SSTR (2a and 5) expression. This case did not differ in its morphologic appearance, revealed no elevated Ki67 proliferative activity, and had a similar immunohistochemical profile compared with those with positive SSTRs.

Normal thyroid tissue is typically negative for SSTR2a; SSTR5 can be very rarely detected in individual cells. The follicular component of strumal carcinoids lacked SSTR2a and SSTR5 expressions, which is in line with former investigations regarding expression of SSTRs in normal tissues [23].

Expression of somatostatin receptors is well known in differentiated neuroendocrine tumors of other origins and is used for diagnostic and therapeutic options [24]. While it remains unclear whether SSTRs are expressed in metastatic strumal carcinoids, there are good reasons from available data from other neuroendocrine neoplasms to assume that STTRs can be expressed in metastatic strumal carcinoids.

Interestingly, NGS analysis of 47 genes (with 2193 amplicons and a total length of targeted regions of 176,370) did not reveal any known pathogenic and/or clinically relevant mutations identified commonly in thyroid carcinoma and neuroendocrine tumors. While the existing solid tumor panel used could not identify possible driver mutations for development of strumal carcinoids, known driver mutations of neuroendocrine tumors (e.g., *PTEN* mutations in pancreatic neuroendocrine tumors or genes of the mTOR signaling pathway in neuroendocrine tumors of the small intestine and the lung [25]) were not detected in this series. Additionally, we could not detect the typical common driver mutations of differentiated thyroid carcinoma genes related to the MAP-Kinase signaling pathway [26], including *BRAF* and *RAS.*

In the literature, malignant behavior of strumal carcinoids seems to be restricted to very few cases. In one malignant case, the primary tumor was found to be calcitonin-positive; however, calcitonin was not found in our series. Additionally, the metastatic tumor exhibited glandular/follicular structures, lacking any markers of neuroendocrine differentiation [9]. Therefore, one may question if the case described represents a true strumal carcinoid. A second report described a bilateral case of strumal carcinoid with metastases originating from the neuroendocrine component. CDX2 immunoreactivity was found both in the putative primary tumors and their metastases [10]. Since CDX2 is generally considered as a marker of intestinal neuroendocrine tumors (we were unable to immune-localize CDX2 in the strumal carcinoids of our series), it also remains questionable whether this case constitutes a genuine metastatic strumal carcinoid or rather represents a case of metastatic (intestinal) neuroendocrine tumor in an ovarian teratoma with thyroid tissue. A third case report described a strumal carcinoid with metastatic spread to the contralateral ovary, the uterine corpus, and the lung. As the contralateral ovary showed similar histology, it was assumed to be metastatic. A potential bilateral occurrence of the disease should also be considered. The metastasis to the uterine corpus was confirmed by histology, showing only neuroendocrine differentiation. Immunohistochemical markers were also not performed both in the presumed primary tumor as well as in metastases. Additionally, the clinically identified lung metastasis was not histologically confirmed [27].

## Conclusion

In summary, strumal carcinoids of the ovary are rare peculiar subtype of ovarian carcinoid tumors with unique features of both thyroid follicular cell and neuroendocrine differentiation, presumably deriving from one precursor cell. While these neoplasms can be diagnosed by their typical morphology and immunohistochemical profile, high frequency of SSTR2a expression may serve as a potential theranostic biomarker in the management of affected patients. While we acknowledge the limitations of NGS panel applied, the absence of common driver mutations in the NGS solid tumor panel may suggest that these neoplasms seem to be genetically unrelated to follicular epithelial–derived thyroid tumors and potentially different than other commonly identified well-differentiated neuroendocrine neoplasms. Therefore, further studies focusing on molecular characteristics of this entity are still needed.
